# Beyond Verbal Behavior: An Empirical Analysis of Speech Rates in Psychotherapy Sessions

**DOI:** 10.3389/fpsyg.2018.00978

**Published:** 2018-06-15

**Authors:** Diego Rocco, Massimiliano Pastore, Alessandro Gennaro, Sergio Salvatore, Mauro Cozzolino, Maristella Scorza

**Affiliations:** ^1^Department of Developmental and Social Psychology, University of Padua, Padua, Italy; ^2^Department of History, Society and Human Studies, University of Salento, Lecce, Italy; ^3^Department of Human, Philosophical and Educational Sciences, University of Salerno, Fisciano, Italy; ^4^Department of Education and Human Sciences, University of Modena and Reggio Emilia, Modena, Italy

**Keywords:** synchrony, referential activity, process research, mixed-effects model, speech rate, paraverbal

## Abstract

**Objective:** The present work aims to detect the role of the rate of speech as a mechanism able to give information on patient's intrapsychic activity and the intersubjective quality of the patient–therapist relationship.

**Method:** Thirty clinical sessions among five patients were sampled and divided into idea units (*N* = 1276) according to the referential activity method. Each idea unit was rated according to referential activity method and in terms of speech rate (syllables per second) for both patient and therapist. A mixed-effects model was applied in order to detect the relationship between the speech rate of both the patient and the therapist and the features of the patient's verbal production in terms of referential activity scales. A Pearson correlation was applied to evaluate the synchrony between the speech rate of the patient and the therapist.

**Results:** Results highlight that speech rate varies according patient's ability to get in touch with specific aspects detected through referential activity method: patient and the therapist speech rate get synchronized during the course of the sessions; and the therapist's speech rate partially attunes to the patient's ability to get in touch with inner aspects detected through RA method.

**Conclusion:** The work identified speech rate as a feature that may help in the development of the clinical process in light of its ability to convey information about a patient's internal states and a therapist's attunement ability. These results support the intersubjective perspective on the clinical process.

## Introduction

The study of clinical processes, due to its complexity and multifactoriality, requires a wide range of points of view and perspectives (Greenberg, 1994; Russell, 1994; Stiles and Shapiro, 1994; Salvatore et al., 2010). In literature, many tools have been developed in order to analyze specific aspects of the clinical psychotherapy process. For example, the innovative moments (Gonçalve et al., 2011), the referential activity (Bucci, 1997), and the two-stage semiotic model (Gennaro et al., 2017). The majority of tools that have been developed focus on psychotherapy session transcripts and the analysis of verbal dimensions (i.e., on explicit or implicit aspects of communication retrieved from semantic and/or syntactical analysis). This, leaves out the deepening of the nonverbal aspects (e.g., patient's and therapist's postures, features of their voices, silences, and so on).

Nevertheless, clinicians acknowledge the main role played by nonverbal communication in the clinical relationship: Kiesler (1979) claims, “the most crucial place to search for relationship is the nonverbal behavior of the interactions” (p. 303). Hall et al. (1995) suggest that the therapist's nonverbal behavior plays a central role in the development of a good clinical relationship. According to Philippot et al. (2003), it plays a role in the development of a good therapeutic alliance. The reliability of such intuitions represent a core aspect in recent years; accordingly several authors focused non-verbal interactions such as body movements (Ramseyer and Tschacher, 2011, 2014), facial expressions (Sharpley et al., 2006), speech disruptions (Horiwitz et al., 1975), tone of voice (Wiseman and Rice, 1989), vocal quality (Tomicic et al., 2011), silences (Frankel and Levitt, 2008), and nonverbal prosodic aspects (Morán et al., 2016). On the whole, results highlighted that non-verbal interaction plays a core role in the development of the therapeutic relationship and its clinical efficacy.

Taking into consideration the different kinds of nonverbal aspects considered in the above mentioned studies, it would be fruitful to differentiate between the nonverbal and paraverbal aspects embedded in communication. Nonverbal aspects are mostly visual (e.g., gestures, head position, global posture, and so on), whereas paraverbal aspects have to do with the quality of the voice (e.g., rate, pitch, volume, speaking style) as well as prosodic features such as rhythm and intonation.

Following Russel (1993), Andersen (1998), Knoblauch (2000, 2005), and Tomicic and Martínez (2011), the paraverbal aspects are regarded as a basic vehicle of nonverbal communication and information exchange in the clinical setting. Paraverbal aspects vehicle implicit meanings (e.g., reflection, anxiety, stress, boredom, etc.) and information about the quality of the clinical relationship (e.g., the presence or absence of synchrony, the similarity or difference in voice tone or other parameters, etc.). Different authors have identified in the coordination or synchrony of adults' dialogic rhythm a key aspect related to the development of the intersubjective clinical attunement (Jaffe and Feldstein, 1970; Feldstein and Welkowitz, 1978; Feldstein, 1998; Cappella and Schreiber, 2006). Attunement can be accomplished through the temporal coordination of microlevel relational behaviors into patterned configurations that become internalized, thus shaping the development of the relationship over time (Rocco et al., 2017).

The present work analyzes speech rate (SR) as a paraverbal aspect. This conveys information about the formal characteristics of patient's intrapsychic activity and the quality of the patient–therapist relationship.

We chose to focus on SR in light of different reasons. Firstly, concerning the technical aspects, the gathering of the data did not require changes to the psychotherapeutic setting. Differing from other paraverbal parameters (for instance, timbre, volume, and others) which require specific tools or patient's positioning, in SR evaluation just a small digital recorder placed anywhere in the therapy room is needed. SR evaluation could be applied either in face-to-face clinical setting, either in classical psychoanalytic setting, (e.g., in the case the patient is lying on the couch). Secondly, SR as paraverbal parameter could be considered, at least from a qualitative point of view, closer to widespread clinical intuitions. On this basis, the clinicians often say “this patient is speaking slower, thus he is not anxious,” or “the patient's speaking faster, which indicates increased anxiety.”

## Paraverbal analysis and multiple code theory

In order to model the role played by SR in the psychotherapy process, we adopted Bucci (1985, 1997, 1999) multiple code theory (MCT) as a framework. MCT takes into consideration the intrapsychic functioning and its manifestation in terms of interpersonal communication. MCT also considers both the explicit (verbal) and implicit (paraverbal) aspects of communication. It analyzes the way these mechanisms participate in the interactive regulation between patient and therapist. Bucci developed her analytical model of the psychotherapeutic process by integrating the constructs of the primary and secondary processes (Freud, 1895) with those derived from studies in cognitive psychology. MCT (Bucci, 2007) identifies two ways of processing information. The first is a symbolic system of comprehending nonverbal (imagery) and verbal (words) as intentional, explicit, and conscious. The second is a subsymbolic system that houses procedural knowledge including the organizing principles of relational repertoires, which are implicit and automatic, mainly operate on an unconscious level (Bucci, 1988), and are mostly nonverbal.

Despite the coexistence of both systems, much of the information exchanged during the therapeutic interaction is manifested tacitly, automatically, and non-verbally (Schore and Schore, 2008). In addition, Bucci (2011) pointed out that the subsymbolic system implies paralinguistic aspects such as tone of voice, intensity, and silences. Bucci affirmed that “paralinguistic features of language may be but are not necessarily connected to symbolic language, and may also carry communicative information in their own channels.” The subsymbolic system is “particularly dominant in emotional communication. Dissonance in communication of emotional meanings occurs when the information carried in the linguistic and paralinguistic tracks do not correspond” (Bucci, 1997, p. 176). Bucci referred to the integration of these systems of information processing as the referential process, a complex cognitive function that could be activated through clinical work. This is to enable the patient to reconstruct those connections between experiences and words that were previously dissociated. The degree to which the referential process has been activated can be measured using the referential activity (RA) method (see Methods section). On the basis of these considerations, Rocco and colleagues (Rocco, 2005, 2008; Rocco et al., 2013) analyzed the relationship between SR and the RA in patients' verbal production. The results highlight that the activation of the referential process reflects a diminishing in patients' speech rate. The higher the referential process, the longer the processing time is, which is due to the more complex process of symbolization (Bucci, 1997). In another work (Rocco et al., 2017), the authors deepened the role played by the synchrony between the SRs of the patient and therapist on the creation of an intersubjective ground. All these studies, that have the limit to refer to a few sessions (from one to three), indicated the role that SR could have in clinical exchanges as a marker of referential process activation.

## Aims and hypotheses

The present work aims to analyze the relationship between the features of the referential process—namely the patient's ability to construct and/or reconstruct the connections between experiences and words—and the impact of these features on the patient's SR during the therapeutic interaction.

We expect that the wider use of a language characterized by formal/organizational features will be reflected in a higher patient speech rate, resulting in a shorter amount of time for speech production. In this case, the referential process would be only partially activated, and less time to produce speech would be needed. On the other hand, we expect that the more the patient uses words connected with subsymbolic, visceral, and evocative contents, the lower the patient speech rate. This results in a longer amount of time for speech production. In this case, the referential process would be strongly activated, and more time to produce speech would be needed (HP1).

As a second goal, we aim to analyze in each session, the process of dynamic synchrony between the SRs of the therapist and patient, assumed as nonverbal expression of the intersubjective clinical attunement coordination (Cappella and Schreiber, 2006). We hypothesize (HP2) that the synchrony will be present as a not steady state across all sessions. This is due to intersubjective attunement which could be represented as the alternation of coordination and miscoordination among clinical courses (Tronick, 1998; Beebe and Lachmann, 2002). So we expect that the stronger the relation between the SRs of the patient and the therapist, the stronger the paraverbal synchrony.

As a third goal, we aim to analyze the relationship between the therapist's speech rate and the features of patients' verbal production, as measured by the referential activity method. We expect that the higher the evocative words used by the patient, the lower the therapist's speech rate (i.e., the therapist's verbal production will be slower). On the other hand, we expect that the higher the formal/organizational terms used by the patient, the higher the therapist's speech rate, reflecting the therapist's ability to acknowledge the patient's difficulty with accessing the subsymbolic dimension in that specific clinical moment, and accordingly using the same speech rhythm (HP3). We expect that the effect of the features of patients' verbal production on therapist's speech rate will appear considering a lagged relationship between the two variables. This is because the therapist needs time to attune to patients' verbal features.

## Methods

### The sample

The present study considers a convenience sample of randomly chosen audiotaped clinical sessions. The total number of sessions was 30, and these were distributed among five psychotherapies. These took place at two psychological services of Padua University from March 2004 to December 2012 (the period of each therapy is reported in Table 1). Each session was entirely audio recorded using a digital recorder placed equidistant between patient and therapist, and entirely analyzed by the method described below. The patient sample was composed of three men and two women (mean age: 31.2; *SD* = 10.57). All patients belonged to middle socioeconomic class. Patients received treatments at a psychological facility in northern Italy. Two of them—patients 1 and 2—received short-term dynamic psychotherapy inspired by Davanloo (1990). This approach emphasizes the importance of active techniques characterized by the use of confrontation and the interpretation of psychological defenses. The approach was integrated by Fosha's (2000) suggestions, concerning the importance of active empathic attention. Patients 3, 4, and 5—received long-term dynamic psychotherapy (weekly sessions, number of sessions ranged from 76 to 218, mean = 156.6, *SD* = 72.9), which was conducted following the theoretical and technical guidelines described by Gabbard (2010). All sessions lasted 50 min each. The therapist was a male who had over 10 years of experience in short- and long-term dynamic psychotherapy, and carried out all therapies. All data were gathered and processed with patients' informed consent.

**Table 1:** DSM V diagnosis, age, job position, kind of psychotherapy, and number of sessions analyzed for each patient.

**Patient**	**Diagnosis DSM V**	**Age**	**Gender**	**Job position**	**Type of psychotherapy**	**Period**	**Number of session analyzed**
1	Erectile disorder	22	Male	Student	Short term	3/2005–7/2005	6
2	Panic disorder without agoraphobia	20	Male	Student	Short term	3/2004–6/2004	1
3	Borderline personality disorder	33	Female	Office clerk	Long term	3/2007–2/2012	5
4	Histrionic personality disorder	46	Female	Office clerk	Long term	1/2008–12/2012	1
5	Schizoid personality disorder	35	Male	Manager	Long term	2/2009–9/2011	17

Table 1 shows the information concerning the patients, the diagnoses, and the therapies.

## Measures and procedures of measurement

### The speech rate (SR) calculation

We measured the SR, by means of specific software named PRAAT, Doing Phonetics by Computer (Boersma and Weenink, 2014) that enables the visualization, annotation, and analysis of sound objects in terms of their acoustic properties, such as frequency, pitch, and time. Transcribed sessions were segmented into idea units (IUs) following the procedures outlined by Bucci and Kabasakalian-McKay (2004; see below for Referential Activity Method). In each IU, the patient's speech rate was calculated as the number of syllables per seconds (as suggested by Auer et al., 1999). A turn at speaking started with the first syllable uttered, continued without interruption, and ended with the last syllable uttered. One or more turns at speaking can be included in each idea unit for both patient and therapist, or for the patient alone. Pauses between the patient's and therapist's turns were not counted in the calculation. Pauses within a turn that exceeded 3 s were treated as 3-s pauses.^1^ The SR was calculated to two decimal points for each idea unit for both therapist and patient. As an example, in the case of the copresence of both patient and therapist speech in the same idea unit (i.e., two turns per IU), SR was calculated as follows. Firstly, the syllables in the patient's turns were summed. Secondly, the number of seconds that the patient spent speaking in each turn was summed. Finally, the number of syllables per unit time was calculated. The same procedure was repeated to obtain patient's speech rate (SRp) and therapist's speech rate (SRt).

In the few moments (never more than four in any given session) when the patient and therapist spoke simultaneously, if the voices were sufficiently clear to permit the technician to understand the moment in which the patient (or the therapist, or both) stopped speaking, then the methodology was applied as described. If the voices were not clear, the technician calculated the SR of the patient (or the therapist, or both) by taking into consideration only the words that could be identified. In Table 2, mean and standard deviation about SRp and SRt are reported (see Results section).

**Table 2:** Example of the database.

**Patient**	**Session**	**Idea unit**	**Patient's speech rate**	**Therapist's speech rate (LAG + 1)**	**CONIM's values**	**CLASP's values**
1	1	1	4.51	NA	1.75	6.50
1	1	2	4.35	6.29	1.75	6.00
1	1	3	4.69	5.74	2.37	6.00
1	1	4	4.15	5.52	1.25	5.25
1	1	5	4.31	5.05	2.00	4.50
1	1	6	4.46	5.40	6.37	8.37
1	1	7	4.95	6.02	4.00	5.87
1	1	8	3.99	6.89	2.00	3.62

*Therapist's SR is highlighted as LAG + 1*.

### Referential activity (RA) method

We measured the referential process by means of the RA Scale (Bucci and Kabasakalian-McKay, 2004), which is used to assess the degree to which a speaker is able to connect verbal and nonverbal representations. These could be by translating somatic experiences, emotions, or representations of actions in words, thus evoking corresponding experiences in the listener. To rate the RA, four scales have been used: Concreteness, Specificity, Clarity, and Imagery. These scales are based on the characteristics of expressive language and are derived from standards used in the psycholinguistic tradition and in literary criticism. According to Bucci (1997), each scale measures a specific feature of language. More particularly, Concreteness indicates the “degree of perceptual or sensory quality, including references to all sense modalities, action, and bodily experience”; Specificity gives information concerning the number of details, such as “explicit descriptions of persons, objects, places, or events”; Clarity reflects how well an image is “seen through the language [and] how well-focused the linguistic image is judged to be”; and finally, Imagery indicates “the degree to which the language evokes corresponding experience in the reader or hearer” (p. 188–189). The mean of the four scales is the global RA value (Bucci, 1997; Bucci and Kabasakalian-McKay, 2004). In light of the scales' intercorrelation, the mean of the Concreteness and Imagery scales (called the CONIM scale) reflects the level of sensory imagery expressed in language. Moreover, the mean of the Clarity and Specificity scales (called the CLASP scale) provides an indicator of discourse organization.

A team of four clinical psychologists (PhD level or trained researchers with previous experience in referential activity application) coded verbatim transcripts of sessions according to the procedure reported in Bucci and Kabasakalian-McKay (2004). As a first step, judges segmented the transcriptions of the sessions into idea units (IUs) and selected boundaries based on the agreement of at least two judges.^2^ The segmentation of the textual corpus produced 1279 IUs. Then, each judge independently rated each IU on all of the four subscales (Concreteness, Imagery, Clarity, and Specificity) on a 10-point Likert scale. For each subscale, IU scores were calculated as the average of the judges' scores. The sum of the values of Concreteness and Imagery concerning a given IU defined the score of CONIM for that IU. The same has been done for Clarity and Specificity with respect to CLASP. Finally, in each IU, the RA was calculated as the average of CONIM and CLASP scales. The interclass correlation coefficient (ICC, Shrout and Fleiss, 1979) was used to estimate judges' interrater agreement for each subscale. The overall ICC for each scale was 0.79 for Concreteness and Specificity, 0.72 for Clarity, and 0.77 for Imagery. In 20 cases, the ICCs were lower than 0.65, indicating fine agreement, and a total of 202 ICCs were higher than 0.80, indicating excellent interrater reliability levels (Fleiss and Cohen, 1973; Fleiss, 1981).

At the end of this process a database was obtained in which, for each Idea Unit (that is our statistical unit) of each session of each patient, the following data was entered: values of patient's speech rate, values of therapist's speech rate + 1 lagged, values of patient's CONIM and values of patient's CLASP. All the variables we considered in this work were time-variant.

Table 2 shows a small example of the database.

**Figure 1:**
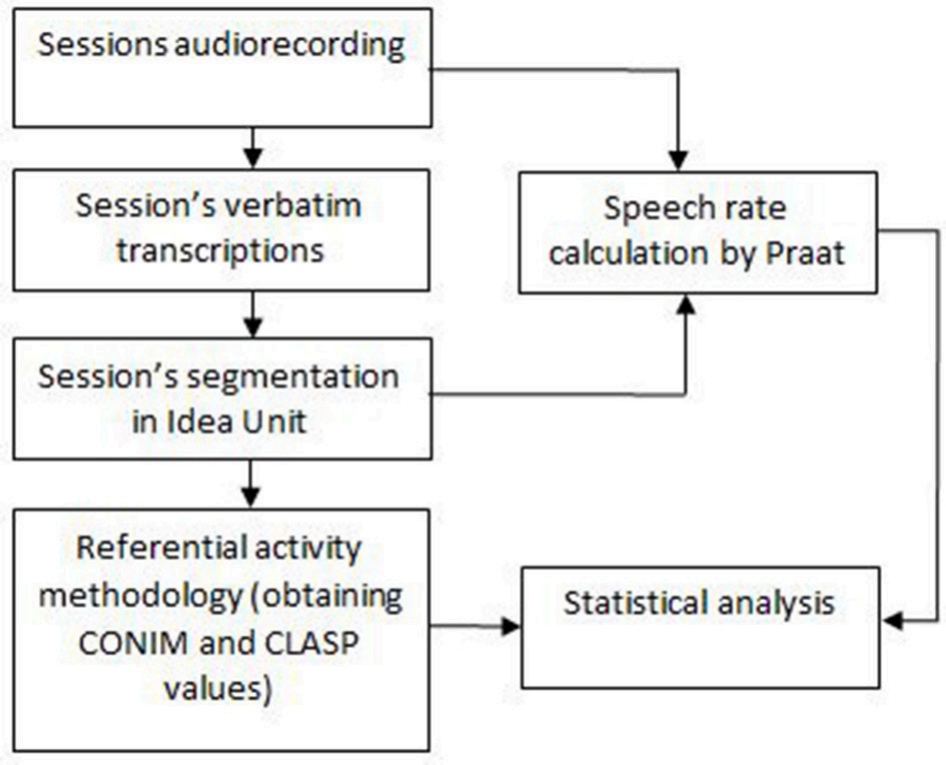
Block diagram describing the procedure of measurements steps to obtain the data.

Figure 1 represent a block diagram reporting the steps followed to obtain the data.

### Data analysis

In order to test HP1 and HP3 separately, we adopted a two-step approach. We estimated the best model performing a model selection approach (Bozdogan, 1987; Myung and Pitt, 1997; Burnham et al., 2011; Fox, 2015). Once the best model was selected, we analyzed the estimated parameters using a Bayesian method (Gelman et al., 2004; Kruschke, 2011).

Specifically, in the first step, we compared a set of mixed-effects models (MEMs; Pinheiro and Bates, 2000) with different fixed effects and with subjects (patients) as random effects (see Brooks and Tobias, 1996).

In the second step, we analyzed the selected model using a Bayesian approach for estimating parameters. In this approach, the uncertainty or degree of belief about parameters values is quantified by prior probability distributions. Then, the observed data are used to update the prior information or beliefs to become posterior information quantified by posterior probability distributions (Gelman et al., 2004; Kruschke, 2011; Lee and Wagenmakers, 2014).

Since the sample (see Table 1) was not homogeneous, a MEM methodology was adopted. MEMs enable estimates to be adjusted (a) for repeat sampling, when more than one observation arises from the same individual, and (b) for sampling imbalance, when some individuals are sampled more than others. Moreover, MEMs allow for variation among individuals within the data (see, for example, Baayen et al., 2002, 2008; Gueorguieva and Krystal, 2004; McElreath, 2016; Borella et al., 2017).

MEMs provide an attempt to quantify the extent to which always-present individual variability predicts variations in the obtained data (Baayen et al., 2008; McElreath, 2016). MEMs were performed to investigate the effect of CONIM (visceral and evocative aspects of verbal production) and CLASP (formal and organizational aspects of verbal production) on speech rate of patients and the speech rate of therapist lagged + 1. First, we considered a null model (m0), including only intercepts and no predictors. Next, we explored the influence of CONIM by adding this predictor (m1). Afterward, we considered the additive model by including CONIM + CLASP as predictors in the model (m2). Finally, we tested the interaction model by including the interaction effect CONIM x CLASP in the model (m3). In each model, we set subjects as random effect.

To compare the aforementioned models, we performed the likelihood ratio test and took into consideration the Bayesian information criterion (BIC; Schwarz, 1978) and the Bayes factor (BF; Morey and Rouder, 2015). The latter allowed us to quantify the evidence of target models compared to the null model where the greater the BF value, the greater the evidence is (Raftery, 1999).

After choosing the best model, as suggested by Rouder and Morey (2012) we sampled the posterior distributions with a Markov chain Monte Carlo (Gelfand and Smith, 1990; Morey et al., 2011; MCMC) process with 10,000 replications. More specifically, by repeatedly sampling from posterior distributions, we were able to produce an empirical approximation of the posteriors (Gelman et al., 2004) and to estimate the parameter values and the highest density intervals (HDIs; Kruschke, 2013)—that is, intervals in which most of the posterior distribution lies, generally the 95%. We adopted the default priors from BayesFactor R-package (Rouder and Morey, 2012), i.e., for regression parameters a normal distribution with mean 0 and variance proportional to a meta-parameter g, distributed as inverse-Gamma function. See (Liang et al., 2008) for more details.

Then, in order to estimate the degree of synchrony between SRp and SRt (HP2), we assessed (Koole and Tschacher, 2016) the synchrony among clinical patterns and calculated the Pearson's correlation. Specifically, in order to evaluate the coordination in temporal patterns, we estimated the correlation between SRp and SRt for each IU from each session.

## Results

Means and standard deviations for number of IUs, the SRs for patients and therapists, sensory information and imagery expressed in language, and the level of formal organization in the discourse were calculated for each patient (see Table 3).

**Table 3:** Descriptive statistics of the five patients.

		**Idea Unit**		**SRp**		**SRt**		**CONIM**		**CLASP**	
**Patient**	**Sessions**	**Mean**	***SD***	**Mean**	***SD***	**Mean**	***SD***	**Mean**	***SD***	**Mean**	***SD***
1	6	24.33	12.91	5.18	1.00	5.21	1.25	2.55	1.27	4.99	1.17
2	1	24.00		4.70	0.42	5.73	0.70	3.19	1.24	5.19	0.82
3	5	28.80	8.41	5.03	0.74	5.16	1.11	2.90	1.71	4.43	1.42
4	1	15.00		5.05	0.36	4.50	0.42	3.57	1.09	5.89	1.02
5	17	23.12	5.81	3.71	0.70	5.35	1.05	3.11	1.22	4.80	1.05

*SRp, Speech Rate of patients; SRt, Speech Rate of therapist; CONIM, level of sensory imagery expressed in language; CLASP, level of discourse organization*.

**Figure 2:**
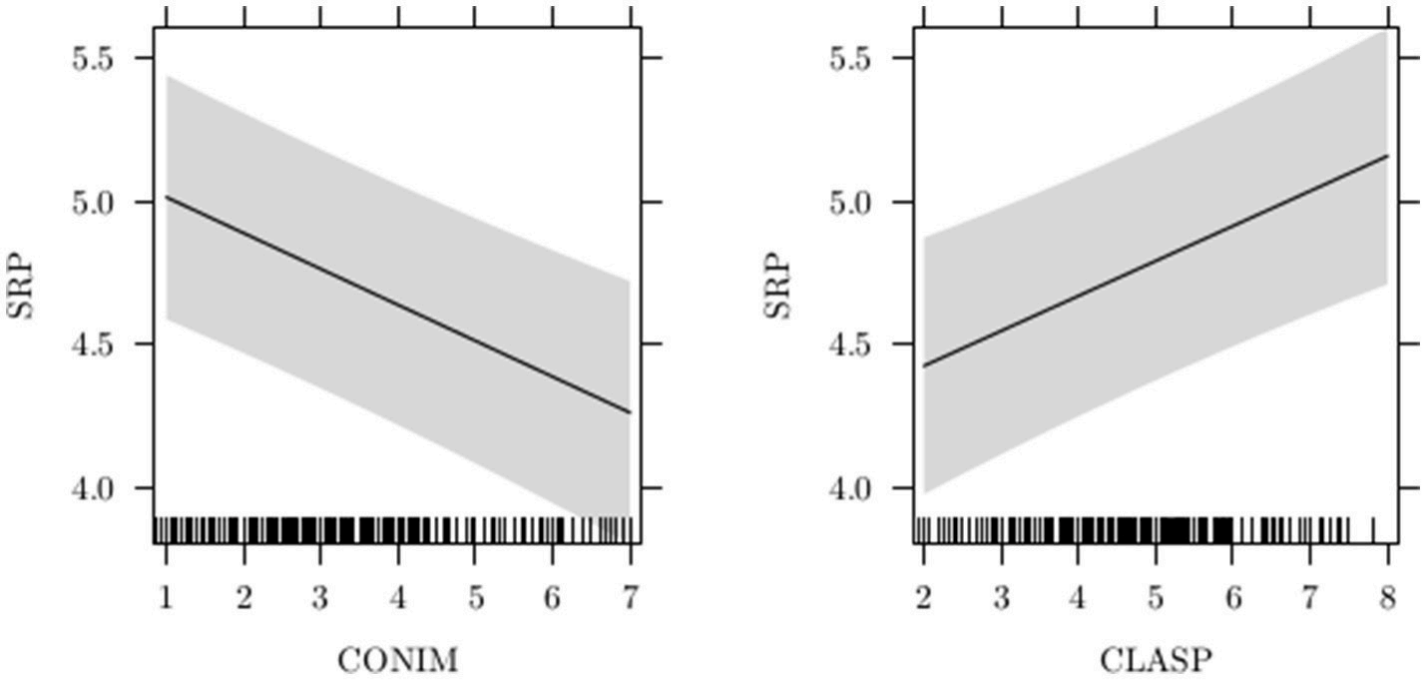
Effects plot for the predictors in model m2. Gray-colored area represents confidence bands around effects (Fox, 2003).

HP1. Table 4 shows the result of model comparisons relative to patients (HP1). The best model was the additive one (i.e., m2 with CONIM + CLASP as predictors), presenting the lower BIC (1614.13) and the higher logBF (141.77). This model appears to be strongly more evident than the null. Note that the difference between model 2 and model 3 was trivial (chi-squared is not significant, BIC difference is 7, and logBF difference is only about 2). Consequently, we considered model 2 the best because it is the more parsimonious model (i.e., has fewer parameters). Figure 2 represents the fixed effects of model m2: SRp decreases by increasing CONIM, while SRp increases by increasing CLASP.

**Table 4:** Fit indices of patients' models.

	**Fixed effects**	**Model df**	**Chisq**	**Chisq df**	***p***	**BIC**	**logBf**
m0		3				1632.69	
m1	CONIM	4	12.73	1	0.000	1626.50	135.04
m2	CONIM+CLASP	5	18.90	1	0.000	1614.13	141.77
m3	CONIM x CLASP	6	0.01	1	0.936	1620.67	139.80

*Df, degrees of freedom; Chisq, chi-squared statistic; p, probability value; BIC, Bayesian information criterion; logBF, logarithm of the Bayes Factor. Patients are the random effect in each model*.

HP2. According to HP2, the general correlation between SRp and SRt was very low (about 0.09). Considering the five patients separately, the values were 0.32, −0.15, 0.22, −0.14, and 0.28, respectively. Moreover, considering the single sessions separately (Table 5), we can note that the correlation values have great variability, varying from high positive values (for example 0.72 in patient 1, 0.85 in patient 5) to negative values (−0.15 in patient 2, −0.12 in patient 5) to null values (−0.05 in patient 3).

**Table 5:** Value of Pearson correlation for different sessions (to be intended in cardinal numbers) of the considered patients.

	**Patients**				
**Sessions (cardinal numbers)**	**1**	**2**	**3**	**4**	**5**
1	0.72^**^	−0.15	0.21	−0.14	0.16
2	0.20		0.15		0.17
3	0.24		0.05		0.38^*^
4	0.13		0.40^*^		0.11
5	0.35^*^		0.30^*^		0.24
6	0.27				0.32^*^
7					0.16
8					−0.12
9					0.85^**^
10					0.13
11					0.43^*^
12					0.28
13					0.31^*^
14					0.17
15					0.21
16					0.40^*^
17					0.52^**^
Mean	0.32	−0.15	0.22	−0.14	0.28

* Medium magnitude effect;

** *Large magnitude effect*.

According to Cohen (1988) operational definition of the magnitude of correlation coefficients, we have 8 sessions with “medium” (≥0.30) magnitude of effect size, and 3 sessions with “large” (>0.5) magnitude of effect size. The other sessions have low or null magnitude effect.

HP3. According to HP3, Table 6 shows the result of model comparisons relative to the SRt. In this case, the effects of variables CONIM and CLASP are small; in particular, the best model is model 1, which has only one predictor (CONIM). This model is quite similar to the null one (logBF is about −5.08), denoting the small effect of CONIM on predicting the SRt.

**Table 6:** Fit indices from therapist models.

	**Fixed effects**	**Model df**	**Chisq**	**Chisq df**	***p***	**BIC**	**logBf**
m0		3				2026.14	
m1	CONIM	4	4.89	1	0.27	2027.75	−0.83
m2	CONIM+CLASP	5	3.52	1	0.061	2030.72	−0.98
m3	CONIM x CLASP	6	0.18	1	0.669	2037.03	−2.59

*Df, degrees of freedom; Chisq, chi-squared statistic; p, probability value; BIC, Bayesian information criterion; logBF, logarithm of the Bayes Factor*.

## Discussion

In this study, the authors deepened the features of a specific aspect of paraverbal behavior (i.e., the SR of both the patient and the therapist) embedded in the clinical exchange. The goal of this deepening, was to test results obtained in previous preliminary studies, within a reduced number of sessions. Results confirmed previous evidences underlying their theoretical and practical role within process research.

### Hypothesis 1

Coherently with the first hypotheses, results confirm the expectations: SRp increases when the formal aspects (subscale CLASP of RA) in patients' verbal productions increase. According to RA method, the increasing of CLASP subscale highlights a partial activation of the referential process and consequently, a partial integration between subsymbolic (i.e., nonverbal and bodily sensation) and symbolic aspects (i.e., words).

Even the second obtained result confirm the expectations: the more the CONIM values grow, the more the SRp decreases. When the CONIM values increase, a bigger amount of the referential process is needed, and because it is a complex cognitive function that integrates subsymbolic and symbolic aspects, it requires a bigger elaboration time. Using Freud's language, it corresponds to a passage of contents from the primary process to the secondary one.

These findings confirm the results obtained in previous studies (Rocco, 2005, 2008; Rocco et al., 2013). Beyond the contents exchanged in psychotherapy communication, implicit aspects of communication can be detected throughout micro-level relational behaviors. Thus, formal characteristics of intrapsychic activity embedded in paralinguistic features represent a further implicit communicative channel. An attuned therapist can unconsciously (or, better, subsymbolically) detect as an expression of the patient's referential process on this basis.

According to this evaluation, the therapist could modulate his interventions. For instance, if the therapist detects, by a decrease in the SR, an integration between subsymbolic and symbolic aspects in a patient's referential process, he or she could evaluate the clinical moment as suitable for an expressive intervention (Gabbard, 2010).

### Hypothesis 2

SRp and SRt correlations change within the course of each session; specifically, the correlation effects vary from null to positive and negative, highlighting effect sizes from low to medium to large (even very large). Such a result suggests that the coordination between patient and therapist SRs represents a non-steady feature in the clinical process.

These results are in line with findings by Tronick (1998), who described the clinical process as an alternation between coordination and mis-coordination moments. In infant research, Jaffe et al. (2001), identified an intermediate model of coordination in mother-child nonverbal interaction; this was characterized by an alternation of optimal coordination and absence of coordination, that can promote flexibility in the child between self- and interactive regulation. In adult treatment, this alternation is part of the principle of “ongoing regulation” between patient and therapist (Beebe and Lachmann, 2002). This ranges from subtle nonverbal behaviors, such as postural and facial interchanges, intonations and tone of voice” (p. 187), and that the authors propose as a process that can promote new expectations and constitute a mode of therapeutic action. In other terms, an intermediate synchrony in patient and therapist SRs could represent a condition allowing the developing of the intersubjective sense-making dynamic paving clinical process (Salvatore et al., 2010). Moreover, such view is consistent with that of Rocco et al. (2017). They considered clinical attunement expressed by paraverbal aspects of communication as features enforcing the therapist–patient relationship and promoting the patient's integration of formal thinking processes affecting emotional and cognitive domains.

### Hypothesis 3

Concerning hypothesis 3, findings only partially confirm the expectation: The more the CONIM values grow, the more the SRt LAG + 1 decreases. It is necessary to underline that the CONIM scale is a moderate predictor of SRt.

A possible explanation of the moderate magnitude of these results could be the following: as therapists well know time is needed for the therapist to enter into the patient's subjective world. This creates an intersubjective ground that can have its expression in a good level of attunement between the patient's verbal production and the therapist's SR. The time necessary to develop this process could impede finding a stronger relationship between the considered variables.

From this point of view, a result in line with our expectation would have been the expression of a therapist that, since the very beginning of each clinical encounter, has been immediately attuned to the patient. This is different from what each therapist experiences in his or her daily clinical activity. As Tronick (1998) said, comparing the mother–infant interaction with the patient–therapist interaction:

The miscoordinated state is referred to as a miscommunication. Miscommunications are normal events. They occur when one of the partners fails to accurately appreciate the meaning of the other's emotional display and in turn reacts inappropriately. The interactive transition from a miscoordinated state to a coordinated state is an interactive repair.…This process can be likened to the process of moving along in therapy. (p. 294).

In conclusion, we cannot exclude the presence, in a single session or part of a single session, of an attunement expressed by the presence of a relationship between SRt and the features of the patient's verbal production. But we cannot expect that this attunement will be present at the point that it could create a statistically significant relationship among the considered variables.

## Conclusion

In this work, we thoroughly explored the role that a specific aspect of paraverbal communication, the SR, plays in psychotherapy. Our aim was to detect which information it can convey about the patient's internal state (in terms of referential process), and its role in describing the quality of therapeutic relationship.

Results confirm that the analysis of the SR of both the patient and therapist enable a wider and deeper evaluation of the complexity belonging to that specific communication system of psychotherapy.

Data highlight that embedded in patients' verbal production, there are, apart from contents, references about the quality of the patient's referential process, a kind of mental activity strictly correlated with the therapeutic process and the therapeutic objectives (Bucci, 1997). We can say that the words, by the medium of their nonverbal and implicit features, provide more awareness about other's internal processes.

Moreover, SRs give us information about the quality of the relationship between the patient and therapist because in some sessions, we see their synchrony/coordination, while in others, it is not present.

This approach to the clinical communicative exchange is similar to the approach used to consider the interaction in infant research, characterized by specific attention paid to nonverbal communication and to a dyadic system view (Beebe and Lachmann, 2002). These authors claimed:

We are both influencing, and being influenced by, our partner's word and actions. Particularly at nonverbal level, mother and child, as well as analyst and patient, participate in a moment-by-moment coordination of the rhythms of behavior. This is the fundamental nature of social behavior. (Beebe and Lachmann, 2002, p. 25).

Later, they added, “Thus, much of the organization of non-verbal communication remains similar across the life span” (p. 26). When applied to the psychotherapeutic context, these aspects of communication are connected to the concept of “implicit relational knowing” (Lyons-Ruth et al., 1998), which should be included in a theory of interaction for psychoanalysis (Beebe and Lachmann, 2002). Our work, placed within this theoretical frame, seeks to propose an integration between the methodology we used and others more devoted to the analysis of content, with the goal of identifying the basic role of development of the clinical process.

## Limitations and future developments

It is necessary to underline that the methodology we used has the limit of being extremely time-consuming; to calculate the SR values of both patient and therapist for a single session of 50 min, about 200 h are necessary.

In this work, only one psychotherapist conducted the clinical sessions we analyzed. To generalize the results, it would be necessary to replicate the methodology in sessions conducted by other therapists. In the available literature, we found only one example of a methodology similar to the one we used (Tonti and Gelo, 2016). Even if the authors are referring to Mergenthaler's therapeutic cycle model (Mergenthaler, 1996, 2008) instead of Bucci's multiple code theory, as in our work, the results are consistent with the ones we found.

In this work, we used the manual RA methodology, which foresees the attribution of RA values by judges. This procedure usually enables obtaining RA values only for patients (Bucci and Kabasakalian-McKay, 2004) because the therapist's verbal production inside each Idea Unit is not always sufficiently wide to calculate its RA values. This limit can be overcome using the computerized RA methodology (Mariani et al., 2013), obtaining therapist's RA values that can be used to further test this hypothesis, and moreover, to add others that should enable confirmation of our basic assumptions.

## Ethics statement

The study was exempt of ethical committee approval since the research work was done only on the tapes of clinical sessions. All the patients included in the research gave their informed consent about the audiorecording.

## Author contributions

All authors listed have made a substantial, direct and intellectual contribution to the work, and approved it for publication.

### Conflict of interest statement

The authors declare that the research was conducted in the absence of any commercial or financial relationships that could be construed as a potential conflict of interest.

## Footnotes

^1^Pauses of 3 s or less within the uttered sentence were included in the measurement of the total time taken to produce the sentence. Pauses exceeding 3 were treated as 3-s pauses. For instance, a pause that lasted 3.8 s was recorded as a 3-s pause. This 3-s cutoff was necessary to avoid SR values being compromised by excessively long pauses that were not due to emotional or cognitive processing but, for example, to crying.

^2^Bucci and Kabasakalian-McKay (2004) define an idea unit as the segment of a patient's speech that captures a single “shot” or “frame” of a narrative. Changes in the mood or feeling of the passage or the introduction of new imagery, shifts in experiential quality, and changes in the scene or person can be used to define the boundary of an IU. Concerning the identification of IU boundaries, Bucci and Kabasakalian-McKay (2004) argued that the knowledge of what an idea is, and where one ends and another begins, is a part of linguistic competence that is shared by speakers of a language and applied intuitively without explicit rules.
